# Shedding Light on Calcium Dynamics in the Budding Yeast: A Review on Calcium Monitoring with Recombinant Aequorin

**DOI:** 10.3390/molecules29235627

**Published:** 2024-11-28

**Authors:** Larisa Ioana Gogianu, Lavinia Liliana Ruta, Ileana Cornelia Farcasanu

**Affiliations:** 1 Doctoral School of Biology, Faculty of Biology, University of Bucharest, Splaiul Independenței 91-95, 050095 Bucharest, Romania; gogianu.larisa-ioana@s.bio.unibuc.ro; 2 National Institute for Research and Development in Microtechnologies, Erou Iancu Nicolae Str. 126A, 077190 Voluntari, Romania; 3 Faculty of Chemistry, University of Bucharest, Panduri Road 90-92, 050663 Bucharest, Romania; lavinia.ruta@chimie.unibuc.ro

**Keywords:** aequorin, calcium monitoring, calcium signaling, *Saccharomyces cerevisiae*

## Abstract

Recombinant aequorin has been extensively used in mammalian and plant systems as a powerful tool for calcium monitoring. While aequorin has also been widely applied in yeast research, a notable gap exists in the literature regarding comprehensive reviews of these applications. This review aims to address that gap by providing an overview of how aequorin has been used to explore calcium homeostasis, signaling pathways, and responses to stressors, heavy metals, and toxic compounds in *Saccharomyces cerevisiae*. We also discuss strategies for further developing the aequorin system in yeast, with particular emphasis on its use as a model for human calcium signaling studies, such as the reproduction of the mitochondrial calcium uniporter. By highlighting previous research and pinpointing potential future applications, we discuss the untapped potential of aequorin in yeast for drug screening, environmental toxicity testing, and disease-related studies.

## 1. Introduction

Calcium ions (Ca^2^^+^) are universal secondary messengers with important roles in a wide array of cellular processes across all eukaryotic organisms. In addition to their functions in stress response and cell cycle regulation in simpler organisms like yeast—which will be reviewed in this paper—calcium signalling is critical in processes such as gene expression [1], autophagy and apoptosis [2,3], and developmental processes [4,5,6]. The regulation of intracellular calcium concentration is achieved through a complex network of calcium channels, pumps, and exchangers alongside intracellular stores like the endoplasmic reticulum and mitochondria [7]. These components enable calcium signals to exhibit remarkable spatiotemporal specificity, such as oscillations or localized calcium sparks, which are essential for their functional outcomes [8]. In mammals, calcium ions mediate physiological activities, including muscle contraction and neurotransmitter release, and disruptions in calcium homeostasis have been studied in relation to various diseases, such as neurodegenerative disorders like Alzheimer’s and Parkinson’s diseases [9,10], cardiovascular diseases [11,12], and cancer [13,14,15]. These pathologies underscore the necessity of precise tools for monitoring calcium dynamics.

*Saccharomyces cerevisiae*, commonly known as baker’s yeast, has long been used as a model organism to study cellular processes due to its eukaryotic nature, ease of genetic manipulation, and well-characterized physiology [16,17]. Although simpler than mammalian systems, yeast cells share many fundamental biological processes with higher eukaryotes [18], making them an invaluable tool for studying calcium signaling pathways. Understanding how yeast cells regulate and respond to changes in intracellular calcium not only sheds light on basic cellular processes but also provides insights into more complex systems, like plant, animal, and human cells. Historically, the measurement of intracellular calcium levels has been a significant challenge due to the dynamic and transient nature of calcium signals. The discovery of aequorin, a bioluminescent protein isolated from the jellyfish *Aequorea* by Osamu Shimomura in the 1960s [19,20], marked a major advancement in calcium research. Aequorin emits light in response to calcium binding, allowing for real-time monitoring of calcium concentrations in biological samples.

The use of aequorin in cellular studies faced significant limitations and was subjected to long-term developments. At first, the need for microinjection to introduce the protein into cells restricted its application to larger cells and specialized laboratories with the necessary expertise [21]. Furthermore, aequorin’s single-photon emission upon calcium binding [22] limited the spatial resolution of calcium measurements, particularly at physiological calcium concentrations where only a small fraction of aequorin molecules is activated. As a result, the use of aequorin declined with the discovery of fluorescent calcium indicators in the 1980s [21,23], which offered easier application and the ability to image calcium signals within cells with greater spatial and temporal resolution. However, the development of molecular biology techniques and the ability to express recombinant proteins in living organisms revitalized interest in aequorin. Recombinant aequorin can be genetically encoded and even targeted to specific cellular compartments, overcoming many of the earlier limitations. This approach has opened new avenues for research, allowing for the study of calcium signaling in a range of cellular processes and under various environmental conditions. Aequorin systems are still widely used and significantly improved today, enabling calcium measurements not only in whole cells but also in subcellular compartments and whole organisms [22,24,25]—aspects that have been thoroughly reviewed in their use in animals and plants [26,27] but not in the budding yeast. Moreover, their applications go beyond fundamental research, as aequorin has also been used as a reporter molecule in biotechnological applications, such as detection assays for small molecules and for in vivo imaging [28].

In this review, we explore the use of recombinant aequorin as a tool for measuring intracellular calcium levels in *S. cerevisiae*. While most research and reviews have focused on the use of aequorin in bioimaging applications in mammalian cells and whole organisms, no less important findings emerged following the expression of aequorin in yeast. This review aims to showcase the advances made in using aequorin to study calcium dynamics in *S. cerevisiae*. We will discuss the genetic engineering strategies used to express and target aequorin in yeast, the applications of this technology in studying calcium homeostasis and signaling pathways, and some methodological considerations that go with these studies. By compiling current knowledge and highlighting recent advancements, this review aims to underscore the value of recombinant aequorin in yeast research and suggest future directions for its application.

## 2. Aequorin: Structure, Mechanisms and Historical Context

### 2.1. Structure of Aequorin

**Figure 1:**
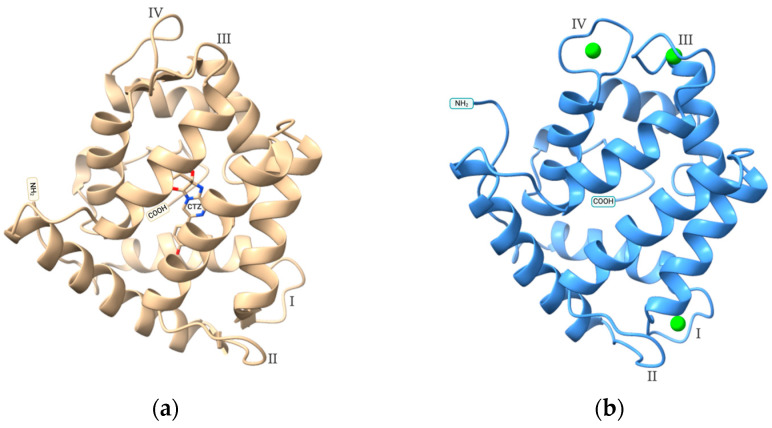
(**a**) Crystal structure of aequorin containing the coelenterazine moiety (RCSB PDB ID: 1EJ3); (**b**) Simulation of Ca^2+^ binding to apo-aequorin using AlphaFold3 [31]. Structures visualization with Chimera X [32] and labeled based on [33]. Roman numerals I–IV indicate the position of the EF-hand loops; CTZ = coelenterazine moiety; green spheres represent Ca^2+^.

Aequorin is a calcium-sensitive bioluminescent protein composed of two key components: the apoprotein (apo-aequorin) and the coelenterazine prosthetic group. Head et al.’s (2000) crystallography study [29] on recombinant apoaequorin and coelenterazine revealed that aequorin forms a globular structure consisting of four EF-hand domains arranged in pairs, with a hydrophobic core cavity that accommodates coelenterazine-2-hydroperoxide (Figure 1a). These EF-hand motifs feature a helix-loop-helix structure, a characteristic of calcium-binding proteins. The EF-hand motifs I, III, and IV are responsible for binding calcium ions and detecting calcium, while EF-hand II does not participate in calcium binding (Figure 1b). Head et al. hypothesized that the EF-hand II might have a role in the enzymatic function of aequorin, as its helices compose part of the walls of the protein cavity and include three amino acids that seem to have a role in positioning the coelenterazine—H58, Y82, and W86. More recent theoretical and experimental studies suggest it has a role in balancing local flexibility and global stability of the aequorin structure, with effects on the luminescence properties; in these studies, replacing the EF-hand loop II with an active form resulted in a faster decay rate [30].

Coelenterazine, the second key element of aequorin, is a small hydrophobic molecule that acts as the bioluminescent substrate for aequorin. Coelenterazine fits into the hydrophobic pocket within apo-aequorin and remains non-covalently bound in its reduced, inactive form. The binding of coelenterazine is necessary for the functionality of aequorin, as it is this substrate that undergoes oxidation during the bioluminescence reaction. This oxidation is catalyzed by calcium binding, leading to light emission, which makes the protein an effective tool for measuring calcium levels in biological systems [20,34].

### 2.2. The Bioluminescence Mechanism

The bioluminescence mechanism of aequorin is activated by the binding of calcium ions to its EF-hand motifs. In its resting state, apoaequorin is bound to coelenterazine, which remains in a chemically reduced form. When calcium ions are present, they bind to the EF-hand motifs of the protein, triggering a conformational change [35]. This structural rearrangement allows for the oxidation of coelenterazine, which is converted into coelenteramide. The oxidation process simultaneously releases a carbon dioxide (CO_2_) molecule. This reaction releases energy in the form of blue light, with a peak emission at approximately 470 nm [36]. The amount of light emitted by aequorin is highly sensitive to calcium ion concentration and exhibits a non-linear dependence due to the cooperative binding of calcium to its high-affinity sites, making it an effective reporter of calcium dynamics [22]. The calcium-binding capacity of aequorin allows it to measure intracellular calcium concentrations in the range of 10^−^^7^ to 10^−^^4^ M, a physiologically relevant range for eukaryotic cells, including yeast [27].

This bioluminescence-based calcium monitoring is advantageous because it does not require external light sources for excitation, which minimizes potential interference with cellular processes. The reaction is considered specific to calcium ions, ensuring accurate measurements of intracellular calcium dynamics. However, early reports have demonstrated that aequorin also reacts with a few other cations, including strontium (Sr^2^^+^), copper (Cu^2^^+^), lead (Pb^2^^+^), and thulium (Tm^3^^+^) [21]. Despite this, these ions are not typically found in high concentrations in living systems, minimizing their potential to interfere with calcium measurements. On the other hand, some studies pointed out that the concentration of Mg^2^^+^ should be taken into consideration when calibrating calcium measurements with aequorin [37].

To convert the raw luminescent signals from aequorin into actual intracellular calcium concentrations, an algorithm that relates [Ca^2+^] to the ratio of L to L_max_, established by Allen and Blinks in 1978, is used [38]. Here, L refers to the light intensity measured under normal physiological conditions (in counts per second), while L_max_ is the total light emitted when all the aequorin in the cells is exposed to a saturating concentration of calcium. To get the most accurate results, it’s important to measure L_max_ at the end of every experiment. This is typically done by treating the cells with a lysis solution to release any unreacted aequorin and expose it to Ca^2+^ [38,39].

The relationship established between [Ca^2+^] and the logarithm of the ratio L/L_max_ allows us to accurately measure calcium levels. When this ratio is plotted on a logarithmic scale, it reveals a straight line within the range of calcium concentrations typically found in cells, which is between 10^7^ and 10^5^ M. This linear relationship allows for straightforward conversion of luminescent signals into precise calcium concentration values. As Grantiero (2013) points out, L_max_ is not a fixed value; it decreases throughout the experiment because aequorin gets consumed. This is why it is compulsory to measure L_max_ at the end of each experiment. After determining this value, the ratio of L/L_max_ can be plotted against a standard curve to estimate the free calcium concentrations in the cytosol. This approach ensures that the measurements are both accurate and reliable [39,40].

### 2.3. Coelenterazine

**Figure 2:**
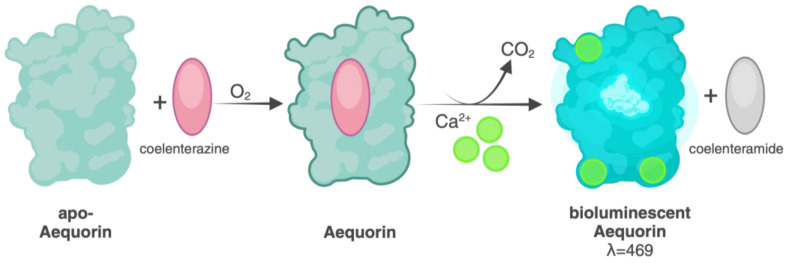
Aequorin light emission upon Ca^2+^ binding. (Created in BioRender. ruta, l. (2024) BioRender.com/v42t872).

Coelenterazine plays a critical role in the functionality of aequorin, acting as the luminescent substrate that undergoes oxidation upon calcium binding. In its reduced form, coelenterazine binds within the hydrophobic pocket of apoaequorin, remaining stable until calcium ions trigger the reaction. When calcium binds to the EF-hand motifs, the subsequent conformational change allows coelenterazine to be oxidized to coelenteramide, accompanied by the emission of blue light (Figure 2).

Shimomura and colleagues were among the first to describe the properties of synthetic analogs of the coelenterazine moiety, which have been used to reconstitute the aequorin complex both in vivo and in vitro [41,42]. These derivatives were named based on their structural modifications, which directly impact their calcium-binding affinities and spectral properties, such as emission wavelength and intensity. For instance, variants like coelenterazine *f* (“fast”) are optimized for rapid responses, while coelenterazine *h* (“high photon count”) produces a brighter signal compared to the native coelenterazine, designated coelenterazine *n*. Such designations reflect each variant’s specific adaptations, offering flexibility for a wide range of experimental setups. While the increased sensitivity of synthetic analogs makes them ideal for detecting small changes in intracellular calcium concentrations, coelenterazine *n* (native) remains suitable for low-sensitivity studies, where reduced light emission is advantageous to prevent over-saturation in high-calcium environments [42]. These properties enable researchers to tailor the aequorin system to specific experimental needs by selecting the appropriate coelenterazine analog. Several of these synthetic analogs of coelenterazine are now widely available for commercial use. For example, coelenterazine *h* can be found in the catalog of several biotechnology companies, including Fisher Scientific (Portsmouth, New Hampshire, USA). Additionally, Biotium (Fremont, California, USA) offers nine different coelenterazine analogs with diverse properties tailored for specific applications.

The membrane permeability [43] of coelenterazine makes it ideal for use in monitoring calcium dynamics in cells, particularly when introducing recombinant aequorin into mutant yeast strains. Its ability to readily permeate cellular membranes enables it to be directly added to the culture medium, from which it diffuses into yeast cells and reaches intracellular compartments containing recombinant aequorin. Once inside, coelenterazine binds to apoaequorin, enabling efficient calcium detection without the need for complex delivery methods. While its permeability simplifies the process, the experimental setup must ensure that sufficient coelenterazine is present to achieve accurate calcium measurements. Since coelenterazine is consumed in the bioluminescent reaction, it may need to be replenished for extended monitoring. 

### 2.4. Historical Development

The discovery and study of aequorin began in the early 20th century with the identification of bioluminescence in the jellyfish *Aequorea*; however, it wasn’t until the 1960s that aequorin was isolated and characterized as a calcium-sensitive bioluminescent protein. The research into aequorin’s calcium-dependent bioluminescence was pioneered by Osamu Shimomura and his research group, who first recognized aequorin’s potential as a tool for calcium measurement [44]. In 1963, Shimomura and his colleagues demonstrated that aequorin could detect low calcium concentrations with higher sensitivity than traditional methods like EDTA titration, making it a valuable alternative for calcium assays. This was exemplified by its ability to measure calcium levels in biological samples such as cow’s milk and horse serum [45]. Building on this initial discovery, Shimomura and Johnson studied further the mechanism of aequorin luminescence. They revealed that the active component of aequorin is a peroxide derivative of coelenterazine, which, upon binding calcium, undergoes a reaction that produces the light-emitting molecule coelenteramide, along with apo-aequorin and carbon dioxide. This reaction highlighted aequorin’s unique role in bioluminescence and calcium detection, setting the stage for its use as a sensitive and selective calcium reporter. By the 1970s, aequorin’s utility in calcium signaling was starting to become apparent, with the new understanding of the nature of the light-emitting reaction and the relation between cations and light emission [36,46].

Despite its early success, the use of aequorin in calcium measurement faced limitations. Early applications required microinjection of the protein into large cells, restricting its use to specialized laboratories capable of handling complex experimental setups. Furthermore, aequorin’s initial versions lacked the spatial resolution needed to visualize subcellular calcium dynamics, providing only an overall light output instead of compartment-specific information. As a result, the scientific community began favoring fluorescent calcium indicators, which offered greater ease of use and higher-resolution imaging. Aequorin, however, experienced a resurgence in the 1980s and 1990s with the advent of molecular biology and recombinant DNA technologies. First, expressed in bacteria, aequorin was more readily available for studies of its structure and properties [47,48]. Second, these innovations later allowed researchers to express recombinant aequorin directly in various types of animal and plant cells and even to target it to specific subcellular compartments [25,49,50,51]. The ability to localize aequorin to distinct cellular compartments reinvigorated its use as a precise calcium indicator, overcoming the limitations of earlier aequorin-based methods. This recombinant approach, combined with its non-invasive use in cells, restored aequorin’s relevance and established it as a powerful tool in modern calcium signaling research. Throughout the 1980s and 1990s, aequorin was expressed in mammalian cells, including human cell lines [52,53], and non-mammalian eukaryotic model organisms like the zebrafish, the fruit fly, and *Dictyostelium* [54,55,56]. The creation of GFP-aequorin fusion proteins in the 2000s further enhanced aequorin’s functionality, improving its sensitivity and allowing real-time in vivo calcium imaging in living organisms [57]. During this period, researchers employed aequorin to study calcium transients during neural activity, muscle contraction, and at various stages in the developmental processes [58,59,60].

In the last decade, even further advancements in aequorin’s utility occurred, particularly with its use in transgenic organisms. Studies employing recombinant aequorin in model organisms enabled researchers to monitor calcium dynamics in real time in various physiological contexts, including neural activity, muscular function, and behavioral responses [61,62,63]. These advances provided new insights into neurodevelopment, muscle diseases, and organismal behavior, proving that aequorin has evolved into a versatile and robust tool in the field of calcium signaling research. Its unique bioluminescent properties and the innovations in its expression and targeting have solidified its position as a fundamental instrument for investigating calcium dynamics in diverse biological systems, from yeast to more complex organisms.

### 2.5. Advantages and Challenges

Despite the emergence of modern calcium indicators like fluorescent dyes or various GECIs (genetically encoded calcium indicators) such as FRET (Förster resonance energy transfer)-based probes and GCaMP (genetically encoded calcium indicators that fluoresce in response to calcium binding), aequorin remains, to this day, a valuable tool in calcium signaling research. Liu et al. (2024) show that despite the emergence of more modern calcium indicators like FRET-based probes or GCaMP, aequorin retains several unique advantages that make it a valuable tool in calcium signaling research [24]. Its non-invasive, real-time monitoring capability, combined with the ability to provide whole-organism or whole-cell measurements, makes aequorin particularly effective for studies requiring broad-scale calcium assessment, such as those examining stress responses. However, the main advantage of using aequorin is that it can be engineered for various purposes, including subcellular localization [64].

Aequorin’s simplicity, cost-effectiveness, and high sensitivity to low calcium concentrations further enhance its utility in high-throughput and physiological studies. However, while aequorin excels in global calcium monitoring, it faces challenges compared to newer methods that offer advantages in subcellular resolution. Techniques like FRET and GCaMP provide enhanced spatial and temporal resolution for calcium imaging within specific cellular compartments, for which choices for detailed studies of localized calcium signalling are sometimes preferred [65,66]. Consequently, researchers must weigh the trade-offs between aequorin’s strengths in whole-cell or organismal studies and the superior resolution offered by newer fluorescence-based methods (see Table 1).

## 3. Recombinant Aequorin

### 3.1. Genetic Engineering and Expression

Several vectors containing the cDNA for the expression of recombinant apoaequorin, using different promoters and selection markers; furthermore, different variants of apoaequorin and fusion proteins have been researched for specific applications, as it will be reviewed in the following.

The apoaequorin nucleotide or amino acid sequence can be freely accessed in databases—e.g., M16103.1 in GenBank and P07164 in UniProt, respectively, for Aequorin 1. The construction of the yeast calcium biosensor implies the transformation of an appropriate yeast strain with a vector containing the DNA for the expression of recombinant apo-aequorin. For this purpose, the sequence of the wildtype apo-aequorin can be used as such, or it can be optimized for each application. The sequence can be minimally twitched for codon optimization—for instance, Sanglard (2021) showed that codon-optimized aequorin serves as a convenient and reliable system for investigating Ca^2^^+^ homeostasis in *C. albicans* [67]—or mutated to improve various aspects of the biosensor, such as thermostability [68,69,70], improved sensitivity or bioluminescence [69,71,72].

Another advantage of the recombinant aequorin is the possibility of coupling it with other recombinant proteins, such as fluorophores (reviewed by Bakayan et al. [33]) localization tags. It is important to keep in mind that the C-terminal proline was found to be essential for bioluminescence emission in aequorin. Therefore, when creating fusion proteins (e.g., GFP-aequorin), the fusion must be attached to the N-terminus of aequorin to preserve its bioluminescent function [73].

### 3.2. Localization and Targeting

While recombinant aequorin can be used as such to measure cytoplasmic calcium levels, it can also be fused with subcellular-specific tags to target aequorin to different organelles for calcium monitoring. A common approach involves fusing the subcellular-specific tag to a GFP-aequorin fusion protein, where the latter combines Green Fluorescent Protein (GFP) with aequorin. This system allows researchers to track both protein localization (via GFP fluorescence) and calcium dynamics (via aequorin bioluminescence), providing a dual signal for enhanced visualization and measurement in specific organelles. Although tagging the aequorin for subcellular localization is a particularly useful tactic for localized calcium measurements, it has mostly been developed for use in animal cells. For example, Ref. [74] Calvo et al. have recently fused the C-terminal of the tetanus-insensitive vesicle-associated membrane protein (VAMP7) to the N-terminal of the GFP-aequorin to confine calcium indicators to the endo-lysosomal lumen in human cell lines. Their system was shown to work for mildly acidic cell compartments, which proves another important advantage of the aequorin system above fluorescent dies: the stability against a wider range of pH values at the measuring sites. Aequorin systems for localized monitoring of acidic organelles in animal cells have been reviewed by Alonso et al. [73] and include variants linked to the Cis- and Trans- regions of the Golgi apparatus (pH 6.7 and 6 respectively), to secretory vesicles (pH 5.5) and to lysosomes (pH 4.5–5). In yeast, however, the application of aequorin fused with organelle-specific tags for targeted calcium measurements is less prevalent compared to other systems, such as mammals. While the use of aequorin in yeast has predominantly focused on cytosolic and mitochondrial calcium measurements, the approach of fusing aequorin with localization tags to monitor calcium in other organelles such as the endoplasmic reticulum, Golgi apparatus, or vacuole remains underexplored.

### 3.3. Optimization of Expression

Directed evolution principles or targeted mutagenesis have been applied to optimize aequorin for enhanced properties (Table 2). Various traits, such as bioluminescence intensity, emission control, faster or prolonged signal, and stability in adverse environments, were the focus of important studies [68,69,70,71,72].

Recently, Haghdoust et al. engineered aequorin to enhance its thermostability by targeting specific residues found through B-factor analysis [68]. They focused on Glu156, identified as a highly flexible residue, and Gly14 in helix A of EF-hand I, a position considered structurally unfavorable. Among the mutants they created, the G14A variant showed an increased thermostability at elevated temperatures compared to the wild type (WT) and a slight increase in bioluminescence. In contrast, the E156N mutation reduced thermostability, while the G14N mutation had no significant effect. Molecular dynamics simulations suggested that the increased stability of the G14A mutant is likely due to a local rise in van der Waals interactions compared to the WT. Meanwhile, the E156N mutation likely caused reduced stability by increasing flexibility in the C-terminal region and disrupting crucial contacts within the protein. Bakayan et al. (2020) needed to overcome the low calcium sensibility of the Redquorin (aequorin fused with a red fluorophore, which is more suitable than GFP for some applications of in vivo imaging) and engineered a variant that outperformed GFP-aequorin [71]; they found that the Q159T mutation increases the sensitivity of the calcium biosensor without a loss of stability compared to the WT aequorin.

## 4. Applications of Aequorin in Yeast Calcium Research

### 4.1. Calcium Homeostasis and Signaling Pathways

Calcium plays an important signaling role in *S. cerevisiae*, as in other eukaryotes, functioning as a universal second messenger involved in processes such as stress responses, mating, and cell cycle regulation. Maintaining calcium homeostasis is essential to ensure that calcium-mediated signals are regulated. Calcium transients are carefully controlled through a network of transporters and channels located in both the plasma membrane and internal membranes, such as those of the vacuole and Golgi apparatus.

In budding yeast, calcium enters the cytoplasm through the High-Affinity Calcium Influx System (HACS), which consists of the Cch1 channel and its regulatory subunit Mid1. This system is activated in response to environmental stresses, such as low calcium or hypotonic shock, allowing calcium influx from the extracellular environment. Additionally, Yvc1, a mechanosensitive TRP-like channel on the vacuolar membrane, mediates the release of calcium stored in the vacuole into the cytoplasm under mechanical stress. To prevent calcium toxicity, the vacuolar calcium pump Pmc1 and antiporter exchanger Vcx1 sequester excess calcium back into the vacuole, while Pmr1 pumps calcium into the Golgi [75,76]. These transporters ensure that calcium spikes are brief and localized, allowing for effective signal transduction while maintaining low cytosolic calcium levels [77,78]. Yeast strains lacking the ion pumps *Pmr1* and/or *Cod1*, both involved in ER/Golgi calcium homeostasis, showed fragmented vacuoles, increased vacuolar calcium uptake, and heightened calcium influx across the plasma membrane, while in *pmr1* mutants, these effects were independent of calcineurin activity, Cch1/Mid1 calcium channels, and Pmc1, but required Vcx1, in *cod1* mutants, increased vacuolar calcium uptake was primarily dependent on Pmc1 and unaffected by Vcx1 deletion [79]. These findings highlight distinct roles for Vcx1 and Pmc1 in vacuolar calcium regulation and suggest the presence of other unidentified calcium influx pathways.

Gdt1 is another Golgi-resident cation exchanger involved in calcium homeostasis, particularly when Pmr1 is absent. Gdt1 plays a compensatory role by controlling the release of calcium from the Golgi into the cytoplasm. This function becomes especially important during osmotic shock, where Gdt1 takes on a major role in managing calcium responses, helping the cell adapt to environmental stress by maintaining intracellular calcium levels [80].

Regulation of calcium signaling in yeast is further modulated by calmodulin (CaM) and calcineurin (CaN). CaM, a calcium-binding protein, activates CaN, a serine/threonine phosphatase that plays a central role in maintaining calcium homeostasis [7]. Activated calcineurin dephosphorylates key targets like Vcx1, fine-tuning its activity and controlling vacuolar calcium sequestration. In addition to this, inositol phosphates generated through the action of phospholipase C (PLC1) play an indirect role in modulating calcium dynamics; although an IP3 receptor has not been properly identified in the budding yeast, mutations in PLC1 affect calcium signaling, indicating that inositol phosphates such as IP3 or its derivatives may regulate calcium release from vacuolar stores, likely through secondary signaling pathways rather than direct channel activation [77,81].

The aequorin system is a go-to technique for fundamental studies of calcium dynamics in *S. cerevisiae*, from calcium channels and pumps to stressors and other elements involved in calcium regulation. This system has been successfully introduced in *S. cerevisiae* for in vitro monitoring of [Ca^2+^] in the cytosol by constitutively expressing recombinant apo-aequorin [82,83]. Nowadays, there are well-documented and detailed protocols, such as the one offered by Tisi et al. [39], which serve as guidelines for replicating experiments and further advancing knowledge in this field. Using aequorin to monitor calcium dynamics, it was found that an increase in extracellular calcium caused a sharp rise in cytosolic calcium, followed by sequestration (into the vacuole) to restore initial levels [84]; this change in calcium levels is followed by alterations in gene expression [85]. Surprisingly, a decrease in extracellular calcium also triggered a cytosolic calcium spike before returning to lower levels [84]. These results suggest the presence of a calcium-sensing mechanism that activates calcium influx channels in response to any external calcium shift, whether an increase or decrease.

The aequorin system introduced in the already-available collection of knock-out mutants for essential genes could represent a good starting point for researching the diverse roles of calcium in cellular processes. By measuring cytosolic calcium levels in mutant strains, researchers can uncover novel elements involved in calcium signaling [86]. It was found, for example, that in cells lacking the calcium/calmodulin-dependent protein kinase Cmk2, a secondary rise in cytosolic calcium takes place after the initial influx, despite the subsequent sequestration of calcium. Cmk2 modulates the activity of Rch1, a protein that inhibits a plasma membrane calcium channel involved in the transport of calcium ions into the cell, such that, without Cmk2, full inactivation of this channel does not occur, leading to prolonged calcium influx [85].

In *ydl206wΔ* mutants, reduced calcium spikes in response to external calcium and pheromone treatments were observed, accompanied by phenotypes suggesting impaired calcium-calcineurin signaling, such as diminished adaptation to mating pheromone and thermal stress [87]. Grx6 is a thiol oxidoreductase protein that was similarly found to have a role in calcium homeostasis in yeast cells. To determine whether cytosolic Ca^2^^+^ accumulation in *Δgrx6* cells correlated with changes in the concentration of calcium at the ER, an assay using an ER lumen-targeted aequorin form was employed. In Grx6-deficient mutants, reduced calcium levels in the ER and increased calcium accumulation in the cytosol led to permanent activation of the calcineurin-dependent pathway [88]. This finding provides evidence that redox regulation plays a role in calcium homeostasis, highlighting the importance of calcium balance for maintaining cellular function in yeast.

Calcium measurements in knockout mutants are also important for understanding the broader functions of calcium in cell signaling. In *akr1Δ* and *erg3Δ* mutants, calcium signaling through calcineurin was shown to regulate intracellular ROS (Reactive Oxygen Species) levels by inducing the expression of *HXT1* and *AGP1*, which are involved in ROS homeostasis and cell survival [89]. This illustrates how calcium helps control gene expression to maintain cellular balance. Additionally, calcium plays a vital role in vesicle trafficking, as shown by a study where overexpression of *SLY41* increased cytosolic calcium, promoting vesicle fusion in the early secretory pathway and compensating for defects caused by the loss of *YPT1*. In this case, calcium enhances the SNARE-dependent fusion step of ER-Golgi transport, restoring proper vesicle trafficking when tethering is impaired [90].

Together, these studies show that calcium measurements in yeast knockouts are key for elucidating the diverse and essential roles of calcium in eukaryotic cells. The next section of the review delves deeper into calcium signaling and homeostasis under stress conditions.

### 4.2. Stress Responses and Environmental Adaptation

Many yeast genes remain functionally uncharacterized, with some likely playing crucial roles in signaling pathways, including calcium signaling. Genetic engineering has become a key approach in uncovering the roles of these genes, and the aequorin system is particularly advantageous for investigating calcium signaling due to its compatibility with mutant studies. The ease with which recombinant aequorin can be introduced into various mutant strains allows for the direct measurement of intracellular calcium level variations on different genetic backgrounds and in response to different stimuli.

One study suggested that the transient elevations of cytosolic [Ca^2^^+^], also known as the TECC response, are primarily driven by calcium influx from the extracellular environment [91]. It showed that a mutant strain unable to phosphorylate glucose did not respond to glucose addition but still exhibited a normal TECC response when galactose was provided. This indicates that hexose uptake and phosphorylation are required specifically for the glucose-triggered TECC response. Furthermore, the TECC response was significantly reduced in low-calcium conditions and completely absent in a *mid1* mutant, which lacks a key subunit of the high-affinity calcium channel in the plasma membrane.

V-ATPase activity, via Vcx1, was shown to be essential for restoring cytosolic Ca^2^^+^ levels after TECC [92]: in wild-type cells, Vcx1 efficiently reduces cytosolic Ca^2^^+^ after a brief pulse, whereas cells lacking V-ATPase activity showed impaired recovery, leading to compromised viability despite minimal changes in overall Ca^2^^+^ levels; notably, *vma2Δ* mutants were better at restoring cytosolic Ca^2^^+^ than concanamycin-treated wild-type cells, suggesting that long-term loss of V-ATPase triggers compensatory mechanisms, primarily through Pmc1p, in a calcineurin-dependent manner. These findings provided important insights into the distinct roles of V-ATPase in calcium regulation. Recent research demonstrated that Ca^2^^+^ influx during the TECC response, observed upon glucose refeeding, is facilitated by membrane hyperpolarization driven by Pma1 (plasma membrane H^+^-ATPase). This study reaffirms Pma1’s role in regulating cytosolic pH and membrane potential and the V-ATPase’s role in organelle acidification [93].

Among compounds shown to induce TECC, salicylic acid seems to mobilize intracellularly stored Ca^2^^+^ [94], whereas PEA (β-phenylethylamine) mobilizes mostly extracellular sources [95].

In studies focusing on the response of yeast to osmotic stress using aequorin as a calcium reporter, distinct patterns of calcium signaling emerge depending on the type of osmotic challenge. Early studies show that hyperosmotic shock induces TECC in *S. cerevisiae*, with a role in adaptation [96]. Batiza et al. [97] demonstrated that hypotonic shock induces a rapid and transient increase in cytosolic calcium, primarily driven by stretch-activated channels; the initial calcium influx derives from intracellular stores, but extracellular calcium sustains the longer phase of the response. Denis and Cyert [98] explored hypertonic shock, revealing that the calcium pulse in response to such stress originates from the vacuole, mediated by the Trpy1 (alias Yvc1) channel, a homolog of the TRP channel family. This result suggests an essential role of this release in adapting to hyperosmotic conditions, whereas extracellular calcium does not play a role in this mechanism. Other TRP homologs have also been linked to hypertonic shock response [99]. *TRPY1* was also shown to be involved in the cellular response to oxidative stress, with increased intracytosolic Mn^2^^+^ enhancing Ca^2^^+^ flux through Trpy1 under oxidative conditions, but not during hyperosmotic shock; this activation is diminished in Mn^2^^+^ transporter mutants (Smf1) and amplified in *pmr1Δ* mutants, linking Mn^2^^+^ homeostasis to Trpy1-mediated calcium signaling [100]. On the other hand, an earlier study shows that oxidative stress primarily induces external Ca^2^^+^ influx, with a more modest contribution from vacuolar Ca^2^^+^ via Trpy1, and does not necessarily involve the Cch1/Mid1 channel [101]. Here, Ca^2^^+^ overload correlates with cell sensitivity to oxidative damage, indicating that excessive cytosolic Ca^2^^+^ exacerbates the stress rather than aiding adaptation.

Aequorin-based calcium monitoring has also been employed to investigate yeast cell responses to metal-induced stress, particularly in relation to heavy metals. The role of Ca^2^^+^ in yeast’s response to various metal stresses, including Mn^2^^+^, Co^2^^+^, Ni^2^^+^, Cu^2^^+^, Zn^2^^+^, Cd^2^^+^, and Hg^2^^+^, was investigated, showing that, notably, exposure to Cd^2^^+^ triggered a sharp increase in cytosolic Ca^2^^+^, with a milder response to Cu^2^^+^, while other metals did not elicit a similar calcium influx [102]. The Cd^2^^+^-induced response primarily relied on external Ca^2^^+^ entering the cell through the Cch1/Mid1 channel, but vacuolar Ca^2^^+^, released via the Trpy1 channel, also contributed. The ability to adapt to high Cd^2^^+^ levels was linked to effective management of Ca^2^^+^ homeostasis, where Cd^2^^+^ tolerance corresponded with strong Ca^2^^+^ pulses, and Cd^2^^+^ sensitivity was marked by a failure to restore low cytosolic Ca^2^^+^ levels, like copper (Cu^2^^+^). Ruta et al. [103] used cytosolic aequorin to further explore how yeast cells respond to high Cu^2^^+^ concentrations. They confirmed that exposure to Cu^2^^+^ led to an increase in cytosolic Ca^2^^+^, a response influenced by both the copper concentration and the oxidative state of the cell and identified the vacuolar Ca^2^^+^ channel Trpy1 as the primary source of the cytosolic calcium increase under copper stress, with additional contributions from Cch1-mediated calcium influx. These findings illustrate the complex interplay between metal stress and calcium signaling in yeast and highlight the value of aequorin in real-time calcium monitoring under such conditions. Calcium fluctuations in yeast cells in response to heavy metal accumulation were further reviewed [104], emphasizing the advantages of using aequorin for monitoring real-time calcium dynamics. Their review highlighted the need for advanced methodologies that can specifically target different cellular compartments, allowing researchers to track calcium changes across multiple phases of the stress response. This compartmentalized approach is essential for understanding the temporal and spatial dynamics of calcium signaling during environmental adaptation.

In addition to metal stress, aequorin has been used to investigate the toxic effects of various natural compounds on yeast cells. Earlier studies show that the antiarrhythmic drug amiodarone showed microbicidal effects by rapidly increasing cytosolic Ca^2^^+^ in *S. cerevisiae* through an influx from outside the cell, which was followed by cell death [105]; this extracellular Ca^2^^+^ influx, similar to responses seen under other stress conditions, involved signaling through calmodulin and calcineurin, which are essential for cell survival. Interestingly, reducing external Ca^2^^+^ or adding high levels of CaCl_2_ helped lessen amiodarone’s toxicity [105]. In contrast, eugenol, a plant-based antifungal, raised cytosolic Ca^2^^+^ by mobilizing both extracellular and vacuolar Ca^2^^+^, yet did not rely on Ca^2^^+^ influx for its toxic effects; unlike amiodarone, eugenol required the Cch1 calcium channel to help cells tolerate its effects, suggesting a unique pathway that could make eugenol useful in combination therapies for drug-resistant infections [106,107]. The toxicity of oleandrin, a potent cardiac glycoside, was studied using cytosolic aequorin in *S. cerevisiae* [108]. It was found that oleandrin exposure caused a significant influx of Ca^2^^+^ into the cytosol, disrupting calcium homeostasis. Yeast strains with defects in vacuolar calcium pumping, which are unable to effectively reduce cytosolic calcium levels, were found to be particularly sensitive to oleandrin. Additionally, it was revealed that oleandrin exposure led to the accumulation of Mn^2^^+^, mediated by the plasma membrane transporter Smf1 and that mutants with impaired Mn^2^^+^ homeostasis were hypersensitive to the compound [108]. These results suggest that oleandrin’s toxicity is linked to both calcium and manganese dysregulation, and manipulating these ion homeostasis pathways may offer strategies for modulating oleandrin’s effects in both toxicological and therapeutic contexts.

Overall, aequorin-based studies have proven essential for understanding how yeast cells adapt to environmental stresses, whether through calcium signaling in response to metal stress, chemical toxicity, or other environmental challenges. This system allows researchers to explore the underlying genetic and physiological mechanisms, offering critical insights into cellular adaptation processes.

## 5. Future Directions

### 5.1. Studies of Mitochondrial Calcium Dynamics

Calcium is an important element for mitochondrial function, including the regulation of metabolic processes and signalling. Although *S. cerevisiae* lacks a native mitochondrial calcium uniporter, its mitochondria can still accumulate calcium under certain conditions. Studies using recombinant aequorin have shown that yeast mitochondria actively accumulate calcium in response to external stimuli, such as ethanol and ionophores, which enhance membrane permeability [109]. However, yeast mitochondria differ from their mammalian counterparts in their calcium handling. For instance, the endogenous calcium transport mechanism in yeast mitochondria is much slower and less effective than the mammalian mitochondrial calcium uniporter (MCU), with transport activities significantly lower than those seen in mammalian cells [109]. Nonetheless, these studies have established yeast as a useful model system for exploring mitochondrial calcium dynamics, especially for examining exogenous transport proteins.

In recent years, the mitochondrial calcium uniporter from humans has been successfully reconstituted in yeast to study its properties in a simpler system. The reconstitution of the uniporter in yeast requires co-expression of the MCU and its essential regulatory subunit, EMRE, to achieve functional calcium uptake [110]. These studies confirmed that the MCU is the pore-forming subunit responsible for calcium transport, while EMRE facilitates its function, as MCU alone is insufficient for activity in yeast mitochondria. Here, fluorescent calcium dyes were utilized to measure mitochondrial calcium uptake instead of aequorin, but these experiments represent the groundwork for drug screening applications. Arduino et al. [111] present a detailed protocol for an accessible MCU-targeted drug screening based on a reconstituted human MCU in in *S. cerevisiae* and the aequorin system for calcium measurements. For this purpose, mitochondria-targeted aequorin is expressed under a strong GPD promoter in yeast cells with mitochondria equipped with the heterologous MCU-EMRE uniporter; the screening is done on isolated and cryopreserved mitochondria, which offer several advantages, such as a minimized false discovery rate due to yeast mitochondria being devoid of an endogenous calcium uniporter and ease of use due to the possibility of storing the engineered mitochondria as a ready-to-use reagent.

The *Plasmodium falciparum* Ca^2^^+^/H^+^ antiporter (PfCHA), typically localized to the mitochondrion in the parasite, was also reconstructed in *S. cerevisiae* but sorted instead to the vacuole; nonetheless, its activity—monitored via aequorin bioluminescence—provides a robust platform for high-throughput pharmacological screening and functional studies of ion transport involving Ca^2^^+^, Mg^2^^+^, and Mn^2^^+^ [112].

### 5.2. Other Emerging Applications

The versatility of aequorin in calcium signalling studies has led to its application in understanding complex biological processes, including those related to human pathology. In studies of Parkinson’s disease (PD), α-synuclein (αSyn) has been shown to disrupt calcium homeostasis. Using the aequorin system, researchers found that expressing αSyn in yeast increased cytosolic Ca^2^^+^ levels and cell death, with both effects significantly reduced by deleting the gene encoding Golgi-resident Ca^2^^+^/Mn^2^^+^ ATPase, *PMR1*. Aequorin measurements further showed that αSyn amplified Ca^2^^+^ spikes in most mutants, except in *pmr1Δ* cells, where this response was inhibited. Deletions of *MID1*, *PMC1*, *VCX1*, and *TRPY1* did not significantly affect the amplification of αSyn-induced Ca^2^^+^ spikes, while deletions of *CCH1* and *COD1* increased the response to external calcium but prevented further spike amplification by αSyn [113]. In a separate study, it was found that *VCX1* deletion altered vacuolar calcium release dynamics, shifting the response from biphasic to monophasic under αSyn overexpression, while overexpression of *PMC1* helped maintain a biphasic release pattern [114]. These studies are good examples of how the cytosolic aequorin system can help clarify calcium-related mechanisms involved in αSyn toxicity, offering insights into PD.

Ca^2^^+^ studies are relevant in advancing drug development, disease treatment, tissue regeneration, and microbial fermentation [115]. It has been suggested that *S. cerevisiae* is an ideal model for investigating calcium signaling proteins linked to diseases, functioning as a living platform to explore the molecular characteristics of heterologous calcium transport proteins or to identify drugs that can restore ion channel function [78,116]. In this context, aequorin offers a valuable tool for monitoring calcium dynamics, which could aid in the design and testing of engineered yeast strains for various biotechnological and pharmaceutical applications. In drug development, yeast can be used for screening of effects such as cytotoxicity or antimicrobial properties, but also for deciphering the mechanisms of action of a particular compound. For instance, experiments on yeast mutants with defects in Ca^2^^+^ transport showed that the cytotoxic effects of *Ailanthus altissima* leaf extracts (AaLEs) were driven by transient pulses of calcium, primarily released from the vacuole into the cytosol [117].

With relevance to medical imaging, lanthanides, commonly used as Ca^2^^+^ channel blockers with important imaging and therapeutic applications, were studied in *S. cerevisiae* to investigate their correlation with toxicity and Ca^2^^+^ channel inhibition, where it was found that lanthanides effectively blocked stress-induced Ca^2^^+^ influx only at high concentrations, with La^3^^+^ and Gd^3^^+^ showing preferential uptake via the Cch1/Mid1 Ca^2^^+^ channel [118]. These findings highlight the potential to control lanthanide accumulation in cells for targeted therapeutic use.

The combination of these approaches underscores the versatility of yeast in both fundamental research and the development of novel therapeutic strategies. The aequorin system, in conjunction with targeted localization and genetic engineering, should be further developed and leveraged to deepen understanding of calcium signalling in health and disease, paving the way for innovative solutions in medical and biotechnological fields.

### 5.3. Potential Improvements of Recombinant Aequorin for Yeast Calcium Research

Aequorin engineering, through directed evolution, aims to improve the protein’s expression, stability, calcium affinity, and emission control, making it more suitable for bioimaging in live cells and whole organisms. Research has identified key areas where mutations can significantly enhance its properties. For example, modifications within the EF-hand calcium-binding motifs, such as F149S, E35G, D117G, and D153G, often alter calcium sensitivity and extend the bioluminescent decay; in contrast, mutations in the coelenterazine-binding pocket, such as Y82F and W86F, shift the light emission spectra, allowing for color adjustment [69,72]. Additionally, mutations like Q168R and L170I improve thermostability, making aequorin more robust in challenging environments, including yeast cells [70].

These insights provide clear strategies for optimizing aequorin for specific yeast-based applications. Slow-decay variants (SloDK mutants) are ideal for long-term tracking of biological processes, as they enable extended signal detection. Spectral tuning through mutations facilitates multiplex imaging, where different emission wavelengths are needed to monitor multiple processes simultaneously. Variants with increased stability in various environments are particularly valuable in industrial applications involving yeast, where strains must perform reliably under stress. By targeting these mutations, aequorin can be fine-tuned for a variety of research and industrial uses.

Recent advancements in aequorin-based methodologies have opened new avenues for studying calcium dynamics across diverse biological systems. For instance, luminescent probes such as GFP-aequorin have been utilized to monitor organelle-specific calcium dynamics, particularly within the endoplasmic reticulum [119,120]. This method provides a robust framework that could be adapted to *S. cerevisiae* to explore calcium flux during cellular stress or metabolic transitions.

Recent studies investigating calcium stress responses using aequorin biosensors include work on plants, such as potatoes, under abiotic and biotic stresses [121] or in *Arabidopsis* during acidic stress [122]. As we have seen, aequorin-based systems have been thoroughly applied to yeast in monitoring calcium stress responses. However, this area of research is not exhausted, as there is still an interest in deciphering the role of calcium in similar signaling responses in yeast exposed to industrial stressors. A series of recent screenings have examined how Ca^2^^+^ signaling enhances yeast’s resilience to high sugar stress, a condition commonly encountered in fermentation processes. In high glucose environments, the presence of Ca^2^^+^ significantly improved yeast growth and metabolic output, including increased ethanol and glycerol production. This effect is linked to Ca^2^^+^-mediated regulation of antioxidant defense mechanisms, such as enhancing the activity of superoxide dismutase (SOD) and catalase and the expression of antioxidant-related genes; moreover, Ca^2^^+^ influences the synthesis of protectants like trehalose, helping yeast cells manage oxidative stress [123]. In addition, it was shown that calcium regulates key metabolic pathways by modulating the activity of enzymes involved in glycolysis, glycerol metabolism, and trehalose synthesis, thus optimizing carbon flow under high-sugar conditions [124]. Furthermore, Ca^2^^+^ signaling stabilizes the expression of genes related to ethanol metabolism and mitochondrial function, ensuring cellular homeostasis and enhanced stress resistance [125]. In the opposite scenario, during glucose starvation, yeast cells release vacuolar calcium into the cytoplasm, triggering a cascade of events that activate the autophagy initiation complex. Specifically, calcium activates Rck2 kinase, which then phosphorylates Atg11, enhancing its interaction with Bmh1/2 proteins and the Snf1-Sip1-Snf4 complex. This leads to the recruitment of Snf1 to the pre-autophagosomal structure (PAS) and the activation of Atg1, ultimately initiating autophagy [126]. These calcium-mediated responses highlight the essential role of Ca^2^^+^ in bridging environmental cues, like nutrient availability, to cellular processes that promote survival under stress.

In complex multicellular systems, aequorin has enabled real-time insights into calcium oscillations and systemic regulation. For example, studies on myocardial contraction in zebrafish larvae [127] and the heart [128] illustrate the tool’s utility in high-resolution calcium dynamics. While these systems differ from yeast, such approaches might inspire studies of calcium regulation in yeast biofilms or multicellular aggregates. Moreover, the use of aequorin in high-throughput receptor assays has been demonstrated in mammalian cells, as seen with the development of a codon-optimized aequorin isotype for GPCR analysis [129]. This indicates potential applications in yeast to study receptor-mediated pathways, such as those involving G-protein-coupled receptors, which are essential for signal transduction in yeast systems.

Incorporating these advancements in aequorin engineering—such as increased sensitivity, improved organelle-specific localization, and refined resolution—could offer deeper insights into calcium signaling in yeast. These improvements would allow for more precise and nuanced studies of calcium dynamics, thereby enhancing our understanding of calcium’s role in cellular processes and homeostasis in *Saccharomyces cerevisiae*.

## Figures and Tables

**Table 1:** Comparisons of aequorin to other calcium measurement methods, including various GECIs and fluorescent dyes, based on [24,64,65,66].

Feature	Aequorin	Comparison to Other Methods
Non-invasive, real-time monitoring	Allows non-invasive, real-time [Ca^2+^] monitoring via bioluminescence.	Require light excitation, potentially causing phototoxicity or interference with cell function
Targeting subcellular compartments	Can be genetically targeted to specific cellular compartments. Can be used for different pH values of the measuring sites.	Offer better resolution for subcellular dynamics.
Whole-cell or whole-organisms’ measurements	Offers a global view of [Ca^2+^] dynamics across the entire cell or even the entire organisms.	Often focused on more localized, subcellular [Ca^2+^] measurements.
Simplicity and cost-effectiveness	Straightforward, suitable for high-throughput studies and screenings, cost-effective.	More complex, requiring specialized imaging systems and more complex setups. Often more expensive.
Sensitivity to low [Ca^2+^]	Sensitive to low [Ca^2+^] ~100 nM.	Sensitive to low [Ca^2+^] ~100 nM.
Quantification	Accurate quantification can be complex due to the single-use nature of the luminescent signal and the fact that coelenterazine is consumed in the process. Requires careful calibration.	Offer easier and more continuous quantification of [Ca^2+^]: e.g., ratiometric measurements enabled by FRET-based probes offer more precise and reproducible quantification across a broader range of [Ca^2+^] over periods of time.

**Table 2:** Effects of some common amino acid residue substitution on aequorin phenotype.

Mutations	Effect	Reference
G14A	Increased thermostability and bioluminescence.	[63]
W86F	Increased thermostability. Shifted emission to shorter wavelengths (466 nm). Increased half-life (t_1/2_ = 3.36 s). Reduced long-term stability.	[64,67]
W86F/D153G	Shifted emission to shorter wavelengths. Reduced half-life.	[64]
D153G	Increased thermostability. Reduced light decay rate.	[64]
Q168R, L170I	Increased thermostability. Increased luminescence intensity	[65]
Y82F	Shifted emission to longer wavelengths (494 nm). Increased half-life (t_1/2_ = 2.91s).	[67]
Y82F/D153G	Shifted emission to longer wavelengths (478 m). Decreased half-life.	[64]
Y82F/W86F	Shifted emission to shorter wavelengths (400 nm). Two-fold increase in half-life.	[64]
Q159T	Best performance when fused to tdTomato (Redquorin), with high sensitivity and unaltered stability compared to wild type.	[66]

## Data Availability

Not applicable.
